# New clinical application of indocyanine green in fluorescence-guided laparoscopic lymph-node biopsy in case of lymphoma. Preliminary results on a case series

**DOI:** 10.1186/s12893-023-02152-x

**Published:** 2023-09-01

**Authors:** Marco Casaccia, Adalberto Ibatici, Filippo Ballerini, Nicolò Maria Barabino, Matteo Santoliquido, Franco De Cian

**Affiliations:** 1 Department of Surgical Sciences and Integrated Diagnostics (DISC), University of Genoa, Genoa, Italy; 2 Surgical Clinic I Unit, IRCCS San Martino Hospital, Genoa, Italy; 3 Haematology and Transplant Centre Division, IRCCS San Martino Hospital, Genoa, Italy; 4 Department of Internal Medicine (DiMI), University of Genoa, Genoa, Italy; 5 Clinic of Haematology, IRCCS San Martino Hospital, Genoa, Italy; 6 Genova, Italia

**Keywords:** Indocyanine green, Fluorescence, Biopsy, Laparoscopy, Lymphoma

## Abstract

**Background:**

Indocyanine Green (ICG) fluorescence-guided surgery is widely used for intraoperative visualization of lymphatic structures. To date, there are no reports indicating this dye being used in lymph node biopsies for suspected or relapsed lymphoma.

**Methods:**

Between October 2021 and June 2022, 12 patients underwent a fluorescence-guided laparoscopic lymph node biopsy (FGLLB) using ICG. The following was retrospectively evaluated: the dosage of ICG, the injection site, the number of patients where fluorescence was obtained after ICG administration, and additionally, the parameters indicating the outcome of the surgical procedure.

**Results:**

The median duration of the surgery was 90 min. A laparotomy conversion was required in one case due to bleeding. Fluorescence was obtained in 10/12 (83.3%) patients by means of subcutaneous/perilesional injection in six of the patients, and intravenously in the other four. Hospitalization had a mean duration of three days. There were no major postoperative complications. FGLLB was used in seven patients to follow lymphoproliferative disease progression, and in five patients to establish a diagnosis. In all cases, FGLLB provided the information necessary for the correct diagnosis.

**Conclusions:**

Fluorescence with ICG offers a simple and safe method for detecting pathological lymph nodes. FGLLB in suspected intra-abdominal lymphoma can largely benefit from this new opportunity which has not yet been tested to date. Further studies with large case series are needed to confirm its efficacy.

## Background

Sentinel lymph node biopsy has been validated as a standard of care in breast cancer, melanoma and gynaecological malignancies [1–3]. With regards to endometrial and cervical cancer, the use of ICG fluorescence imaging has been extensively investigated and has proven to be a safe, time-efficient and reliable method compared to standard tracers such as the Patent blue and technetium 99 m [4].

In the gynaecological field, recently published systematic reviews recommend the use of ICG as the tracer of choice for most gynaecological cancers [5].

However, the use of this dye in primary lymph node diseases has not yet been sufficiently studied. To date, there are only a few anecdotal reports indicating its use to detect pathological lymphatic tissue when a lymph node biopsy is performed for suspected lymphoproliferative disease [6, 7].

Taking advantage of the known properties of an elective tropism for the lymph node tissue of the ICG, the decision was made to study the use of this fluorophore for the intraoperative identification of pathological lymphatic tissue. In case of abdominal lymph nodes affected by primary lymphoproliferative diseases, the hypothetical advantage, since not yet fully studied, would be to simplify the search for the lymph nodes themselves and to be able to choose the biopsy site with greater precision by targeting the vital tissue needed to obtain a contributory pathological examination. Thus, a prospective series of patients undergoing a fluorescence-guided laparoscopic lymph node biopsy (FGLLB) to rule out, or to follow the progression of a lymphoma, was studied.

## Methods

**Fig. 1:**
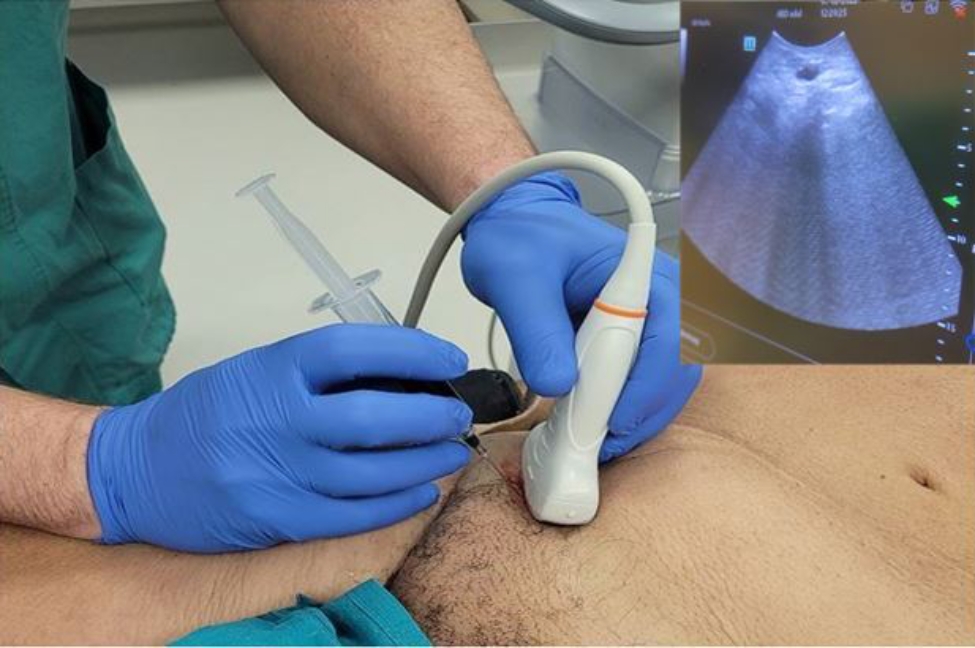
Intra lymph node injection of the ICG solution at the groin under ultrasound guidance

In October 2021, FGLLB was introduced for the first time in our division. By October 2022, twelve procedures had been performed and collected in a prospective database. The clinical characteristics of the patients included sex, age, previous abdominal surgery, and associated comorbidities. The purpose of the biopsy was to establish a diagnosis or confirm the progression of lymphoma. All patients had intra-abdominal lymphadenopathy and an absence of lymphadenopathy accessible to a superficial biopsy. The pre-operative work-up included a computer tomography (CT) scan and a positron emission tomography (PET) for all patients. During the laparoscopic procedure a solution obtained by diluting a 25 g vial of indocyanine green powder (Verdye, Diagnostic Green GmbH, Aschheim-Dornach, Germany) with 10 ml of sterile water was used. The concentration of the solution to be injected was kept constant for all patients in the study. The site of administration of the ICG dye varied with the anatomical site of the specimen to be retrieved. In case of inframesocolic lymph nodes (ILN) biopsy, the injection of 2 mL of the aforementioned solution was carried out in each of the inguinal regions, in the subcutaneous/intra lymph node position (Fig. 1). In case of supramesocolic and mesenteric lymph nodes (SLN) biopsy, a 2 mL injection was carried out in the peritoneum in the vicinity of the pathologic lymph nodes (i.e., perilesional).

**Fig. 2:**
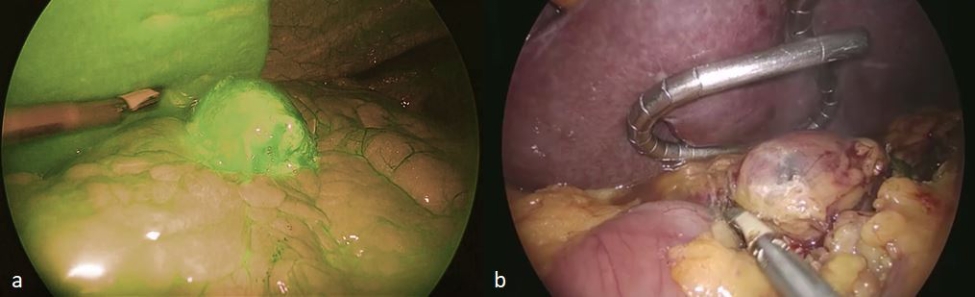
(**a**) Pathologic celiac lymph node fluorescence after intravenous injection of ICG solution. (**b**) Lymph node retrieved “in toto” by means of Thunderbeat dissection

In case of no lymph node staining through these routes, a “rescue procedure” was carried out by means of an intravenous administration of the solution previously described at the dosages normally used for this route of administration (0.5 mg/kg), followed by the evaluation of the fluorescence and subsequent lymph node excision. (Fig. 2).

The indicators of the procedure outcome taken into consideration were: the operative time, the estimated blood loss, the associated interventions, the surgical conversion, the insertion of additional trocars, the positioning of an abdominal drain, the length of hospital stay and the post-operative complications. To evaluate the effectiveness of the use of the ICG in fluorescence-guided surgery, the drug dosages, the injection site of the drug and the number of patients with fluorescence obtained after ICG administration were also considered.

### Surgical technique

**Fig. 3:**
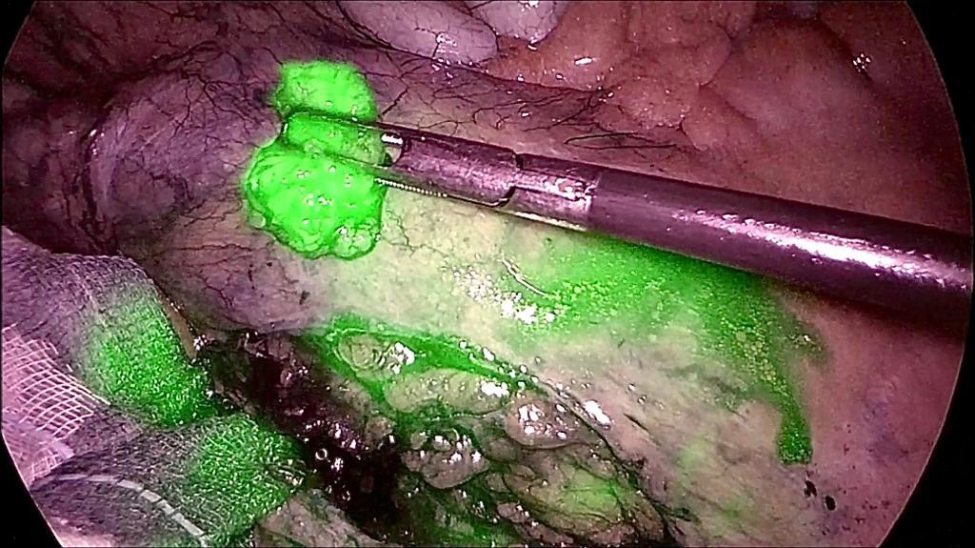
After peritoneum incision, a peri-iliac lymph node is retrieved under fluorescence guidance

In the case of ILN biopsy, after induction of anaesthesia, 2 mL of the aforementioned ICG solution was injected subcutaneously at both inguinal regions, at the level of the femoral triangle of Scarpa. The pneumoperitoneum was achieved through the umbilicus via an open technique; a 10-mm trocar was inserted to accommodate a 30°-angle telescope. A 10-mm trocar was inserted in the right iliac fossa and a 5-mm trocar in the right hypochondrium. The surgeon and camera holder stood on the right side of the patient. A right tilt and mild Trendelemburg position were given to the table. Taking advantage of the lymphatic drainage that first drains to the external iliac lymph nodes from the superficial inguinal lymph nodes, then to the pelvic lymph nodes, and finally to the paraaortic lymph nodes twenty minutes after the injection, observations were made of the lymph nodes stained with ICG in the subserosa along these vessels. Para-aortic lymphadenopathy was reached by an incision of the peritoneum covering the superior part of the right common iliac artery. This incision was guided along the left aspect of the aorta, just above the inferior mesenteric artery. A Thunderbeat™ (Olympus Medical Systems Corp., Tokyo, Japan) device was used for dissection. A dedicated clinical endoscopic system (Visera Elite II, Olympus Medical Systems Corp., Tokyo, Japan) equipped with infra-red (IR) light source and IR UHD telescope was used to illuminate regional lymph nodes (Fig. 3).

In case of SLN biopsy, the surgeon stood between the patient’s legs and a mild reverse Trendelemburg position was given to the table. The camera was inserted at the umbilicus and two further trocars were added at the right and left flank. The injection of the IGG solution was made in the perilesional peritoneal fat through a catheter equipped with a needle introduced through a trocar.

Surgical intervention for all patients consisted of a laparoscopic biopsy of lymphatic tissue. Small lymph nodes were retrieved “in toto” realizing an excisional biopsy; if the pathologic tissue was in form of lymphomatous plaque due to the fusion of multiple lymph nodes or in the case of pathological extranodal lymphatic tissue, an incisional biopsy was carried out.

## Results

The gender ratio was six males to six females. The patient median age was 67 years (range: 38–77 years). Median BMI of the patients was 26.3 (range: 20.4–38.6). Previous abdominal surgery was present in five patients. FGLLB was completed laparoscopically in 11 cases. A laparotomy conversion was required in one case due to bleeding and an abdominal drainage was left in place in this patient. Generally, three trocars were needed, however, two patients needed a supplementary trocar to better expose the field. The estimated blood loss was absent in all patients except in those converted from laparoscopy to laparotomy.

The abdominal lymph nodes sampled were ILN (periiliac and periaortic) in nine cases and SLN (coeliac trunk and gastrohepatic ligament) in three. The biopsy was excisional in seven cases, incisional in three cases and both methods were used in two cases. Fluorescence was obtained in 10/12 (83.3%) patients by means of subcutaneous/perilesional injection in six of the patients and intravenously in the other four. No fluorescence was noted in cases of perilesional injection. The clinical features are detailed in Table 1.

**Table 1:** Appearance of lymph node fluorescence after injection in different sites

	ILN (n = 9)	SLN (n = 3)	Overall (n = 12)
Sites of injection			
Subcutaneous injection (%)	6/9 (66.6)	NCO	6/9 (66.6)
Perilesional injection (%)	NCO	0/3	0
Intravenous injection (%)	2/3 (66.6)	2/3(66.6)	4/6 (66.6)
All the sites	-	-	10/12 (83.3)

ILN, Infra mesocolic lymph nodes; SLN, supra mesocolic lymph nodes; NCO, not carried out

Median duration of surgery was 90 min (range: 60–120 min). Length of hospitalization was 1.5 days (range: 1–11 days). Morbidity was null.

A prolonged postoperative stay of 11 days was due to the development of complete ureteral obstruction as a result of tumoral compression needing a nephrostomy and a stent. A preoperative positron emission tomography/computed tomography (PET/CT) scan showed solid lymphomatous tissue enveloping the terminal portion of the subrenal abdominal aorta and the left common iliac artery.

In seven patients FGLLB was used to follow the progression of a lymphoproliferative disease, and in five patients to establish a diagnosis. The histopathological diagnosis was non-Hodgkin lymphoma in nine patients, a specific inflammatory reaction in two patients and metastasis from an undifferentiated solid tumour in one case, respectively. In each retrieval of the 9 cases where a non-Hodgkin lymphoma diagnosis was obtained, the samples were multiple and all were positive, both when the sampling was incisional and when it was excisional. In all cases FGLLB provided adequate specimens of lymphatic tissue and a correct diagnosis with subsequent therapeutic decisions being achieved.

## Discussion

The subdivision of the abdominal lymph nodes sites involved in the laparoscopic biopsy essentially reflects the different pathways of the lymphatic flow. In fact, for the ILNs the injection of the dye took place in the inguinal region. However, this route of administration is not effective for the SLNs since the paths of the lymph flow are more complex.

Unfortunately, for the SLNs an injection in the regions adjacent to the lymph nodes proved to be ineffective in staining the lymph nodes and in two out of three cases the staining was obtained only after the intravenous injection, i.e., with the “rescue procedure”.

The subcutaneous injection at the groin certainly proved to be more effective in cases of ILN. In fact, in six out of nine cases, the para-iliac and periaortic chains were coloured which made the sampling easier. In the remaining three cases, fluorescence was obtained in two, and only thanks to the “rescue procedure” of intravenous dye injection. It should be noted that in these last three cases the disease was in the form of a large tumour mass and of extra lymph node origin.

Extranodal involvement can be seen with lymphoma in approximately 25–40% of cases and almost any organ can be involved [8]. This could explain the lack of vital staining as extra-nodal lymphomatous tissue is excluded from lymphatic drainage, and could further limit the dye’s usefulness in detecting pathologic lymph nodes. However, in our case, it was mandatory to perform a biopsy on the largest lesion since it had the highest SUV and was, therefore, able to provide us with the best diagnostic indications [9].

Another peculiarity that was observed during laparoscopic biopsy of pathologic ILNs was the possibility that lymph nodes greatly enlarged due to the disease constitute a “stop” to the up flow of the dye by preventing its entry into the lymph node itself and into the lymphatic chain immediately downstream.

The issue of whether the dye is retained by the pathological lymph node in the same way as it is in healthy ones is currently unknown. In colo-rectal or gastric cancer, after the peritumoral injection of ICG, the dye follows the lymphatic pathways and colours the lymph nodes it finds along the collectors. The presence of cancer cells within the metastatic lymph node does not seem to decrease the intensity of staining [10].

However, this statement is not univocal. Widespread lymph node metastases have been observed to induce an obstruction of lymphatic channels, and lymphatic drainage is bypassed to other (nonsentinel) lymph nodes [11]. Perhaps a possible solution would be to inject the dye 24 h before the procedure to give it a chance to come up even if the flow is pathologically slowed. In fact, when lymph node mapping such as that performed in gastric surgery must be performed, a submucosal peri-tumoral injection of the dye takes place 24 h prior through an endoscopic route. In these cases, visualization occurs because of the persistence of the dye even long after the injection [12].

## Conclusions

In conclusion, the issue whether the elective tropism of the dye towards the lymph nodes is maintained in cases of primary lymph node disease has been proven. Concerns remain about the ideal dosages of ICG and the injection sites. A potential advantage to be investigated is the ability, thanks to the dye, to carry out the surgical sampling on a defined vital area, avoiding false negatives due to the sampling of necrotic tumours or acellular fibrous areas, thus improving the diagnostic accuracy. Furthermore, in the case of abdominal lymph nodes affected by primary lymphoproliferative diseases, ICG-enhanced fluorescence seems to provide several advantages in FGLLB by allowing both to better identify the surgical anatomy and to potentially reduce the surgical time due to a safer dissection. Further studies with large case series are needed to confirm these promising data.

## Data Availability

The datasets generated and analysed during the current study are available in the IRCCS San Martino Hospital repository. Responsible person to contact: Tisa Valentino, tel. +39-010-555 7643.
